# Supplementary figures and images for: Elucidating the mechanistic association of xylene inducing non-small cell lung cancer through network toxicology and molecular docking analysis
Source: PLoS One. 2026 Mar 13;21(3):e0341548. doi: 10.1371/journal.pone.0341548 (PMC12987433; doi:10.1371/journal.pone.0341548)

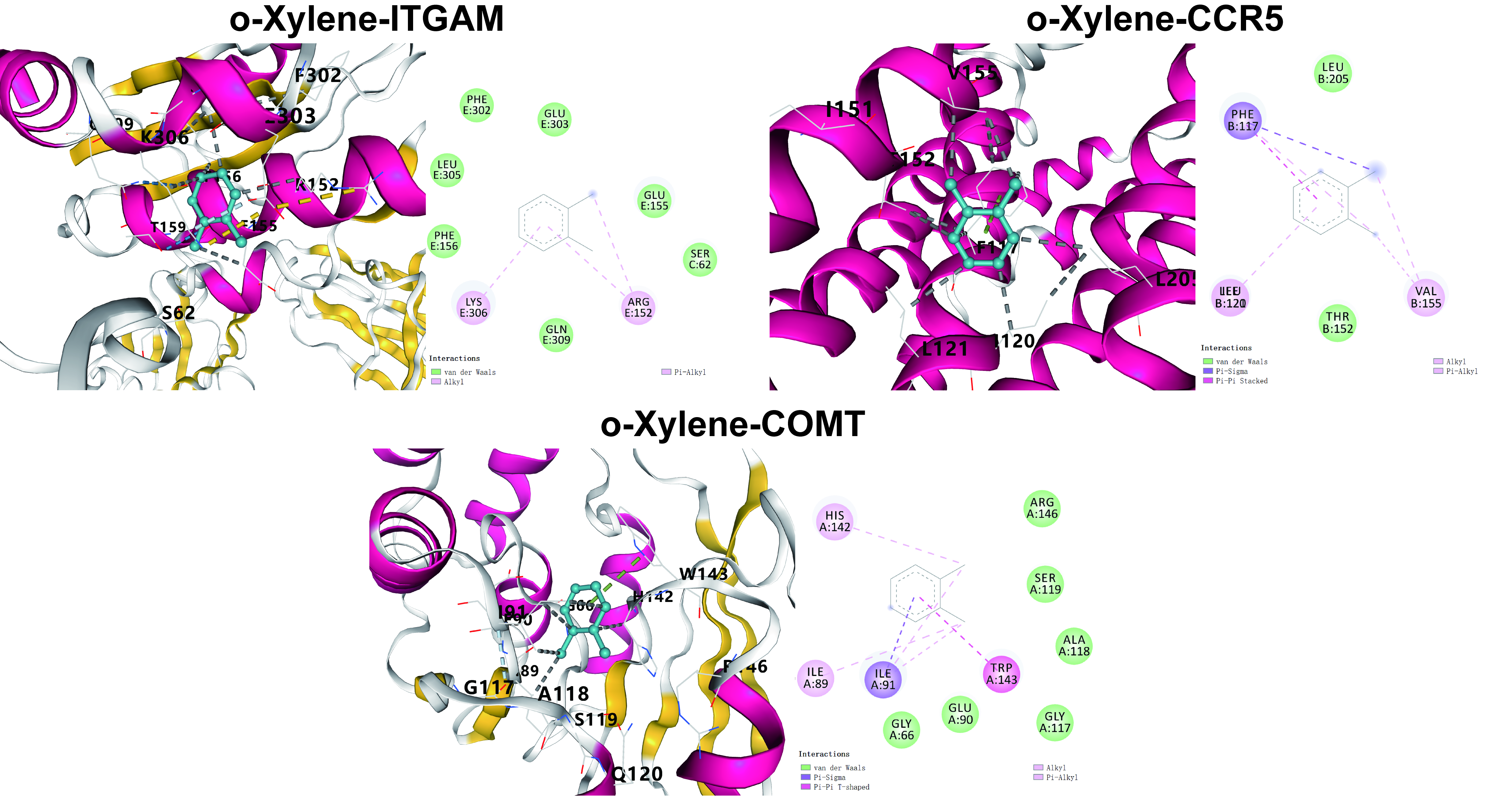

Supplement: S1 Fig — (TIF) [file pone.0341548.s001.tif]

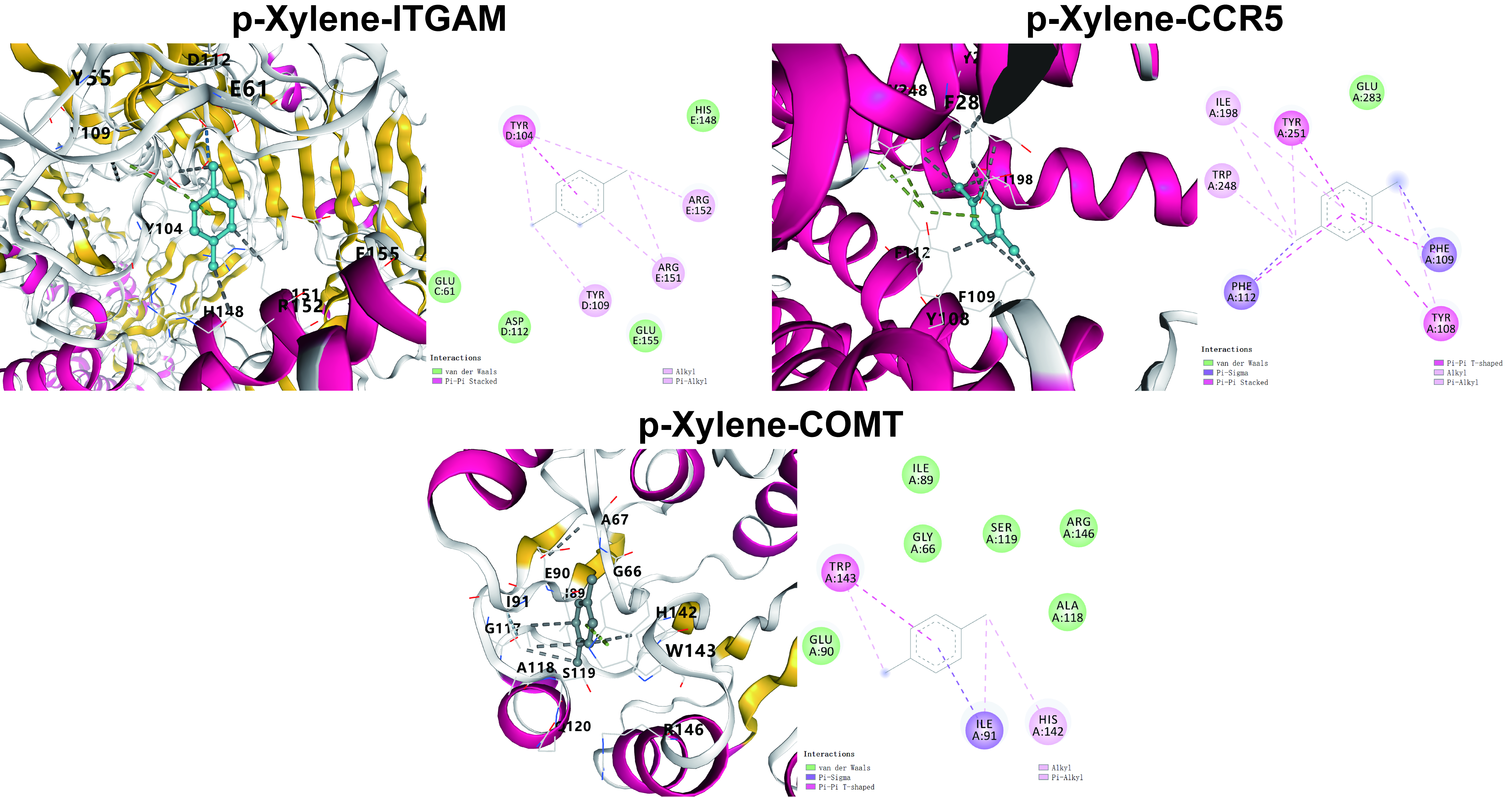

Supplement: S2 Fig — (TIF) [file pone.0341548.s002.tif]
