# Supplementary material for: Elucidating the mechanistic association of xylene inducing non-small cell lung cancer through network toxicology and molecular docking analysis
Source: PLoS One. 2026 Mar 13;21(3):e0341548. doi: 10.1371/journal.pone.0341548 (PMC12987433; doi:10.1371/journal.pone.0341548)
Supplement: Attachment — Submitted filename: Review Report_PONE-D-25-53598.pdf [file pone.0341548.s006.pdf]

This manuscript explores the potential mechanistic link between environmental xylene exposure and the development of non-small cell lung cancer (NSCLC), using network toxicology, enrichment analyses, scRNA-seq data reanalysis, and molecular docking simulations. The topic is timely and relevant, given the growing evidence of environmental contributions to lung cancer, especially among non-smokers. The study is ambitious in integrating multiple computational approaches and identifies five candidate targets (IL1A, H3C13, ITGAM, CCR5, and COMT) potentially mediating xylene-induced carcinogenesis. However, while the manuscript is informative, several methodological limitations, gaps in validation, and issues with presentation need to be addressed before the work can be considered for publication.

Major concerns:

1. The study is entirely computational. While the predictions are interesting, the absence of *in vitro* or *in vivo* validation significantly limits the biological relevance. Without experimental confirmation, conclusions should be framed more cautiously. Phrases like *"confirmed strong binding affinities"* or *"xylene may disrupt important signals and promote tumor growth"* are overly conclusive and too strong for computational predictions. At minimum, authors should validate gene expression patterns of the identified core targets using publicly available NSCLC datasets (e.g., TCGA, GEO bulk RNA-seq).

2. The process of intersecting xylene-related targets with NSCLC-associated genes may yield false positives due to database biases. It is unclear how the authors ensured specificity. Please provide more details on the filtering criteria (e.g., confidence scores, interaction thresholds) and include a sensitivity analysis.

3. The scRNA-seq dataset used (GSE198099) is relatively small (two patients and two controls). This limits generalizability. H3C13 was not detected in the dataset, yet it is still considered a "core target." Authors should clarify whether this undermines its relevance.

4. Docking scores alone cannot confirm biologically relevant interactions. The binding energies reported (–4.8 to –6.3 kcal/mol) suggest moderate affinity but not necessarily strong biological activity. Please include control compounds or positive ligands for comparison to contextualize the docking results.

5. Some conclusions (e.g., xylene may polarize macrophages or promote immune escape) are speculative without functional data. The discussion should be toned down to reflect hypotheses rather than established mechanisms.

6. While the study claims translational significance, it does not clearly connect predictions to potential preventive or therapeutic strategies. The reviewer would suggest expanding the

discussion on how findings could inform biomonitoring, exposure regulation, or drug development.

Minor concerns:

1. Several sections require English polishing for clarity and conciseness (e.g., “xylene due to its high volatility and potential for bioaccumulation may play a critical role…” could be simplified).

2. Figures are informative but lack sufficient resolution for evaluation. Ensure high-quality images are provided. Legends should be more descriptive (e.g., specify thresholds used in enrichment analysis).

3. While many references are recent, some are not directly supportive of specific claims. Ensure all references appropriately match the statements made.

4. Provide exact database access dates for reproducibility. Clarify statistical thresholds (e.g., adjusted p-values for enrichment analysis).

5. The ethics statement says, “not applicable,” but scRNA-seq data from human samples were used. The authors should clarify whether the dataset had prior IRB approval and if their reanalysis was exempt.

6. Literature review on xylene-specific carcinogenic mechanisms is limited; more background on known genotoxic/epigenetic effects of xylene would strengthen rationale.

7. The criteria for selecting 115 intersecting targets are unclear. Were thresholds applied to avoid weak/indirect associations? No discussion of false positives from database mining. Only degree centrality (via cytoHubba) was used in PPI networks and core targets. Multiple topological measures (betweenness, closeness) would provide more robust selection.

8. Adjusted p-values (FDR correction) should be reported in enrichment analysis. Current reporting of only $p < 0.05$ may inflate significance.
