# Supplementary material for: Elucidating the mechanistic association of xylene inducing non-small cell lung cancer through network toxicology and molecular docking analysis
Source: PLoS One. 2026 Mar 13;21(3):e0341548. doi: 10.1371/journal.pone.0341548 (PMC12987433; doi:10.1371/journal.pone.0341548)
Supplement: Attachment — Submitted filename: Response to Reviewers.doc [file pone.0341548.s008.doc]

Dear Editor,

We would like to express our sincere gratitude to you and the reviewers for the insightful comments and valuable suggestions provided during the review of our manuscript titled "Elucidating the Mechanistic Association of Xylene Inducing Non-Small Cell Lung Cancer through Network Toxicology and Molecular Docking Analysis".

We have carefully considered all the feedback and have made extensive revisions to improve the quality and clarity of our manuscript. Below, we provide a detailed response to each of the reviewers' comments and outline the specific changes made to the manuscript.

**Response to Major Comments:**

**Comment 1:** The study is entirely computational. While the predictions are interesting, the absence of in vitro or in vivo validation significantly limits the biological relevance. Without experimental confirmation, conclusions should be framed more cautiously. Phrases like “confirmed strong binding affinities” or “xylene may disrupt important signals and promote tumor growth” are overly conclusive and too strong for computational predictions. At minimum, authors should validate gene expression patterns of the identified core targets using publicly available NSCLC datasets (e.g., TCGA, GEO bulk RNA-seq).

**Response 1:** We fully agree with the reviewer's point that computational predictions require cautious interpretation and need experimental support to establish biological relevance. Accordingly, we have made the following key additions in the revised manuscript: First, as suggested, we validated the expression patterns of the core targets using public databases (TCGA and GEO, GSE19188); second, we further provided in vitro evidence through RT-qPCR experiments in lung cancer cell lines; and finally, we have carefully revised overly conclusive statements throughout the text (for example, changing “confirmed strong binding” to “suggested potential binding”) and explicitly framed mechanistic speculations as hypotheses to be tested.

**Comment 2:** he process of intersecting xylene-related targets with NSCLC-associated genes may yield false positives due to database biases. It is unclear how the authors ensured specificity. Please provide more details on the filtering criteria (e.g., confidence scores, interaction thresholds) and include a sensitivity analysis.

**Response 2:** We thank the reviewer for raising the important issue of database bias. To enhance specificity, we implemented a multi-step filtering strategy and conducted the suggested sensitivity analysis. First, to mitigate false positives, we aggregated targets from complementary sources (xylene: ChEMBL/STITCH, retaining only high-confidence interactions; NSCLC: GeneCards/OMIM, focusing on established disease genes). Common targets were identified after strict UniProt ID standardization. Second, we applied a medium-confidence threshold (interaction score ≥0.4) in STRING for initial PPI network construction. For the resulting network (107 nodes, 334 edges), key hubs were identified by applying rigorous median topological thresholds: Closeness ≥ 0.028, Betweenness ≥ 25.27, and Degree ≥ 6.447. Third, we performed a sensitivity analysis by reconstructing the network under a stringent high-confidence threshold (≥0.7). Under this stricter condition, the same five core targets (IL1A, H3C13, ITGAM, CCR5, COMT) remained among the top-ranked nodes. This demonstrates that their identification as key hubs is robust and not dependent on a single, more lenient cutoff.

**Supplementary Figure:** Protein-protein interaction network of the intersecting targets generated using the STRING database with a high-confidence interaction score threshold of ≥0.7.

**Comment 3:** The scRNA-seq dataset used (GSE198099) is relatively small (two patients and two

controls). This limits generalizability. H3C13 was not detected in the dataset, yet it is still considered a “core target.” Authors should clarify whether this undermines its relevance.

**Response 3:** We fully agree with the reviewer's points regarding the limited sample size of the scRNA-seq dataset and the non-detection of H3C13. Indeed, this prompted us to apply a stricter cross-platform consistency filter. Within our validation framework, H3C13 was not detected in both the GEO dataset and this scRNA-seq data, which contradicts its upregulation observed in the TCGA database and cellular models. This suggests its expression may be highly context-dependent. Consequently, based on consistent expression patterns across platforms, we ultimately identified ITGAM, CCR5, and COMT, which show a stable downregulation trend in TCGA, GEO, and cellular experiments, as the core potential targets associated with xylene exposure. We have updated this conclusion in the manuscript and have discussed the platform-dependent discrepancies observed for H3C13 and IL1A.

**Comment 4:** Docking scores alone cannot confirm biologically relevant interactions. The binding energies reported (–4.8 to –6.3 kcal/mol) suggest moderate affinity but not necessarily strong biological activity. Please include control compounds or positive ligands for comparison to contextualize the docking results.

**Response 4:**  We thank the reviewer for this important and constructive comment. We fully agree that docking scores alone are insufficient to confirm biologically relevant interactions, and that comparison with control compounds or known ligands would provide valuable context. In this study, our primary aim was to conduct a preliminary computational screening to identify potential interactions between xylene and our core targets derived from multi-omics analyses. The molecular docking serves as a hypothesis-generating step to prioritize targets for future wet-lab experimental validation (e.g., competitive binding assays, functional studies), which we have now more clearly stated as a necessary future direction in the revised manuscript. At this stage, identifying a universally accepted “positive control” small molecule ligand with confirmed biological activity for each of these specific protein targets (ITGAM, CCR5, COMT) in the context of xylene exposure is challenging, as such reference data are limited in the literature. To address the reviewer’s concern about overinterpretation, we have significantly toned down the language in the results and discussion. We have replaced definitive claims of “strong binding” with descriptions of “potential interaction” and have emphasized the preliminary and predictive nature of the docking results, clarifying that they require experimental confirmation. We acknowledge that incorporating control comparisons is a strength for rigorous docking studies. As suggested, this will be a key component in our follow-up experimental work aimed at functional validation.

**Comment 5:** Some conclusions (e.g., xylene may polarize macrophages or promote immune escape) are speculative without functional data. The discussion should be toned down to reflect hypotheses rather than established mechanisms.

**Response 5:** We fully agree with the reviewer's comment. In the revised Discussion section, we have explicitly reframed statements regarding xylene's potential effects on macrophages or the immune microenvironment from conclusive inferences into testable hypotheses based on the available data, with a clear emphasis on their predictive nature and the necessity for future experimental validation.

**Comment 6:** While the study claims translational significance, it does not clearly connect predictions to potential preventive or therapeutic strategies. The reviewer would suggest expanding the discussion on how findings could inform biomonitoring, exposure regulation, or drug development.

**Response 6:** We appreciate the reviewer’s suggestion. In the revised Conclusion, we added a concise section outlining how ITGAM, CCR5, and COMT may inform biomonitoring in exposed populations, guide refinement of exposure standards based on genetic susceptibility, and suggest potential avenues for future drug or preventive strategy development. These additions strengthen the translational connection of our findings.

**Response to Minor concerns:**

**Comment 1:** Several sections require English polishing for clarity and conciseness (e.g., “xylene due to its high volatility and potential for bioaccumulation may play a critical role…” could be simplified).

**Response 1:** Thank you for the reviewer's suggestion. We have simplified and rewritten the relevant sentences in the original text in accordance with your comments to make the expressions more direct and clear. For instance, the original sentence "xylene due to its high volatility and potential for bioaccumulation may play a critical role..." has been revised as follows: "Xylene is a common environmental and occupational pollutant, may contribute to lung cancer development due to its high volatility and tendency to accumulate in the body." Similar revisions have been made throughout the entire manuscript to enhance overall readability.

**Comment 2:** Figures are informative but lack sufficient resolution for evaluation. Ensure high-quality images are provided. Legends should be more descriptive (e.g., specify thresholds used in enrichment analysis).

**Response 2:** In response to this comment, we have re‑generated all figures in high‑resolution formats (minimum 600 PPI) to ensure clarity and revised all figure legends to include explicit methodological details. For example, enrichment‑analysis figures (e.g., Fig. 3) now clearly state the specific statistical thresholds applied (adjusted p‑value < 0.05). All modifications are highlighted in the revised manuscript for ease of review.

**Comment 3:** While many references are recent, some are not directly supportive of specific claims. Ensure all references appropriately match the statements made.

**Response 3:** Thank you for this important comment. We have carefully checked the alignment between all statements and their supporting references in the text. Inaccurate or indirect citations have been corrected, and the revised manuscript now ensures that every claim is appropriately referenced.

**Comment 4:** Provide exact database access dates for reproducibility.Clarify statistical thresholds (e.g., adjusted p-values for enrichment analysis).

**Response 4:** We thank the reviewer for these critical suggestions. In the revised manuscript, we have now provided the exact access dates for all used databases (e.g., TCGA, GEO, PubChem) and explicitly stated all statistical thresholds, including the adjusted *p-value* (p.adjust) < 0.05 applied in the enrichment analyses. These additions are clearly indicated in the Methods section and corresponding figure legends to enhance reproducibility and clarity.

**Comment 5:** Provide exact database access dates for reproducibility.Clarify statistical thresholds (e.g., adjusted p-values for enrichment analysis).

**Response 5:** We thank the reviewer for the important comment on ethics. All data in this study, including scRNA-seq data, were sourced from publicly available, de-identified repositories (GEO and TCGA). As our work involved only the re‑analysis of such existing public data, it was exempt from additional ethics approval per institutional guidelines.

**Comment 6:** Literature review on xylene-specific carcinogenic mechanisms is limited; more background on known genotoxic/epigenetic effects of xylene would strengthen rationale.

**Response 6:** Thank you for this suggestion. We have added content in the introduction on the potential carcinogenic mechanisms of xylene, along with supporting references, to strengthen the background rationale.

**Comment 7:** The criteria for selecting 115 intersecting targets are unclear. Were thresholds applied to avoid weak/indirect associations? No discussion of false positives from database mining. Only degree centrality (via cytoHubba) was used in PPI networks and core targets. Multiple topological measures (betweenness, closeness) would provide more robust selection.

**Response 7:** We thank the reviewer for these important suggestions. To address the specificity of the 115 intersecting targets, we have clarified our multi-source filtering strategy (as detailed in response to Major Comment #2) and acknowledged potential false positives in the Discussion. Furthermore,We have enhanced the methodological description by specifying the use of multiple centrality measures in the PPI network analysis. Key hubs were identified by applying the following median topological thresholds: Closeness ≥ 0.028, Betweenness ≥ 25.27, and Degree ≥ 6.447. This multi‑metric approach strengthens the robustness of core target selection.

**Comment 8:** Adjusted p-values (FDR correction) should be reported in enrichment analysis. Current reporting of only p < 0.05 may inflate significance

**Response 8:** Thank you for this correction. We have updated the manuscript to explicitly report the FDR-adjusted p-values (p.adjust) for all enrichment analyses (GO and KEGG) in both the Methods section and the corresponding figure legends (Fig. 3). The revised text now states that significance was defined as p.adjust < 0.05, replacing the previously generic p < 0.05 reporting.

We have updated the Funding statement to include an additional grant number (2025J01686) from the Natural Science Foundation of Fujian Province, which supports the present study. The revised funding information has been corrected accordingly in the manuscript.

We sincerely hope that this revised manuscript has addressed all your comments and suggestions. We appreciated for reviewer’ warm work earnestly and hope that the correction will meet with approval. Once again, thank you very much for your comments and suggestions.

Best regards,

Yours sincerely,

Hongquan Chen and Yuanlin Qi

School of Basic Medical Sciences, Fujian Medical University, Fujian, China, 350122

Email: 13843202849@163.com and ylqi@fjmu.edu.cn

Phone: 86-13843202849 and 86-15005081308
